# A Functional Biological Molecule Restores the PbI_2_ Residue-Induced Defects in Two-Step Fabricated Perovskites

**DOI:** 10.3390/molecules28207120

**Published:** 2023-10-17

**Authors:** Yuanmei Huang, Guoping Yu, Danish Khan, Shuanglin Wang, Yujie Sui, Xin Yang, Yu Zhuang, Jun Tang, Huaxi Gao, Ming Xin, Abuduwayiti Aierken, Zeguo Tang

**Affiliations:** 1 School of Energy and Environment, Yunnan Normal University, Juxian Road 768, Chenggong, Kunming 650500, China; 2 College of New Materials and New Energies, Shenzhen Technology University, Lantian Road 3002, Shenzhen 518118, China; 3 College of Materials Science and Engineering, Beijing University of Technology, 100 Pingleyuan, Beijing 100124, China

**Keywords:** two-step method, PbI_2_ residue, artemisinin, perovskite solar cells

## Abstract

Coating the perovskite layer via a two-step method is an adaptable solution for industries compared to the anti-solvent process. But what about the impact of unreacted PbI_2_? Usually, it is generated during perovskite conversion in a two-step method and considered beneficial within the grain boundaries, while also being accused of enhancing the interface defects and nonradiative recombination. Several additives are mixed in PbI_2_ precursors for the purpose of improving the perovskite crystallinity and hindering the Pb^2+^ defects. Herein, in lieu of adding additives to the PbI_2_, the effects of the PbI_2_ residue via the electron transport layer/perovskite interface modification are explored. Consequently, by introducing artemisinin decorated with hydrophobic alkyl units and a ketone group, it reduces the residual PbI_2_ and improves the perovskites’ crystallinity by coordinating with Pb^2+^. In addition, artemisinin-deposited perovskite enhances both the stability and efficiency of perovskite solar cells by suppressing nonradiative recombination

## 1. Introduction

A growing number of researchers and industrialists now believe that perovskite solar cells (PSCs) are on the verge of commercialization as the power conversion efficiency (PCE) gears up to an excellent level, i.e., 26.1% [1]. It is an unusual outlook at a time when other thin-films emerging photovoltaics are expensive or not comparatively efficient, and the PCE in conventional silicon solar cells is saturated at a specific value. Although the commercialization of PSCs seems interesting to lower the energy conversion cost, long-term stability, and high PCE via industrially available fabrication techniques, it is a sine qua non. In this virtue, depositing perovskite via an anti-solvent approach is the main barrier to scalable perovskite layers [2]. Therefore, a two-step method, where two different precursors are prepared, i.e., for the BX_2_ (generally PbI_2_) and AX (organic salts) layers, are deposited sequentially to engineer the perovskite (ABX_3_) [3]. Nevertheless, the mechanism of perovskite formation in the two-step method is more complicated than the one-step method, owing to the interface formation between the PbI_2_ and organic salts [4]. More specifically, the physicochemical characteristics of both the lead and organic halides govern the perovskite formation [5]. Organic salt’s evaporation and incomplete penetration in the PbI_2_ bulk leave behind the unreacted PbI_2_, which generally remains as a residue in the final perovskite layer [6]. Although the moderate amount of PbI_2_ within the grain boundaries is beneficial, owing to its passivation effect [7,8,9], its excessive amount suppresses the carrier transportation near the perovskite interfaces, especially under illumination [10,11]. Therefore, controlling the amount of excess PbI_2_, especially at the perovskite interfaces, can boost the PSCs’ performance through suppressing the nonradiative recombination [12,13].

Several additives have been applied to the PbI_2_ layer, which enhanced the PSCs’ performances by passivating the Pb^2+^ defects. For example, Boopathi et al. introduced alkali metal halides in the PbI_2_ layer and improved the PbI_2_ film’s compactness by passivating the Pb^2+^ [14]. In 2015, Zhang et al. incorporated an additive (4-tert-butylpyridine, TBP) to create a porous PbI_2_ to enhance the organic salt penetration in the PbI_2_ layer [15]. Zhao et al. used carbonyl group-based monomers as an additive in the PbI_2_ precursor, improving the interactions between the carbonyl groups and the Pb^2+^ [16]. Furthermore, Wang et al. partially substituted Pb^2+^ with neodymium cations (Nd^3+^) by doping PbI_2_ with Nd and achieved a PCE over 21% [17]. In more than 90% of cases, the additives are introduced in the PbI_2_ precursor during the two-step perovskite method, and in each of these cases, the additives provide Lewis acid passivation to the Pb^2+^ defects. Nonetheless, the reason behind the additive importation in the PbI_2_ part, rather than salt precursors, was ill-defined until Liang et al. examined the Pb^0^ defects in detail and concluded that the Pb^0^ defects are induced by the PbI_2_ layer under illumination. They further proved that the Pb^0^ impurities are decomposition byproducts of residual PbI_2_ [18]. Very recently, Yang et al. also found that the Pb^2+^ and Pb^0^ defects are abundantly present at the residual PbI_2_ sites [19].

Herein, the Pb^2+^ passivation effect at the interface of the SnO_2_ and PbI_2_ layer is studied, where the chances of PbI_2_ residue are comparatively high [20]. As on the amines’ deposition at PbI_2_, the perovskite conversion stops right after the resultant wall formation in the form of the perovskite layer, further inhibiting the penetration of organic cations. Comparatively, this perovskite barrier formation is more critical in FA-riched organic cations where the PbI_2_ with FAI reaction is fast [21,22,23,24]. Consequently, a large amount of PbI_2_ residue is generated in the FA-based perovskite layers beneath the perovskite. This work uses FAI-rich amine precursors, and the SnO_2_ substrate is decorated with a functional biological molecule, artemisinin (ART). On the ART interaction with the resultant perovskite, the interface defects, especially the Pb^2+^ defects, are strongly hindered. Furthermore, the amount of PbI_2_ residue is decreased in the final perovskite film. As a result, PCE is enhanced from 20.21% to 21.72%, mainly due to the improvement in the FF and V_OC_ of the device. Commercially available ART has a unique molecular structure composed of sesquiterpene lactones surrounded by alkyls [25], which makes it hydrophobic, and its ketone group provides the Lewis base interaction and passivates the Pb–I antisite defects [26]. Previously, it was used as an additive of perovskite a couple of times, where it successfully reduced the Pb^2+^ defects and improved the PSCs’ performances [27,28]. Nevertheless, to the best of our knowledge, we are studying it as a passivation layer at the defective interface of the SnO_2_/perovskite to control the PbI_2_ residue and Pb^2+^ defects for the first time.

## 2. Results and Discussions

**Figure 1:**
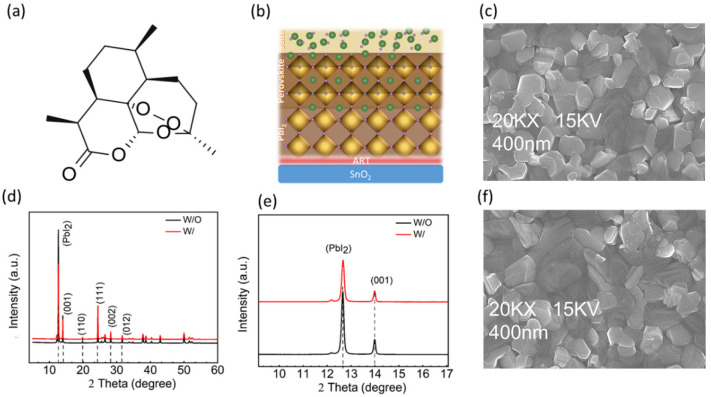
(**a**) Molecular structure of ART; (**b**) schematic of perovskite formation in two-step method; (**c**) top SEM of control perovskite film; (**d**) XRD full spectra of the control; (W/O) and ART incorporated (W/) perovskite films (**e**) PbI2 peaks in W/ and W/O ART perovskite; and (**f**) top SEM of ART-based perovskite film.

Molecular structures of small molecules or polymers have their specific effect on the performances of PSCs when used as additives or in the contacts of the perovskite layer [29,30,31]. However, tedious experimental synthesis and reproducibility remained a question mark for these organic materials. Therefore, commercially available and cheaper organic materials can pave the commercialization. ART is a cost-effective organic biological molecule with a ketone group (C=O) (Figure 1a) that enhances the Pb^2+^ interaction and passivates the Pb-I antisite defects [32]. ART is spin-casted on the SnO_2_ layer, which sandwiches it as a SnO_2_/ART/perovskite, as shown in Figure 1b, where it has maximum interaction with the perovskite region and, moreover, this interface has a high concentration of PbI_2_ residue. In the two-step fabricated perovskite layers, the Pb^2+^ defects increase due to the PbI_2_ residue [18,19]. Therefore, there are more chances that the unreacted PbI_2_ is clustered at the SnO_2_/perovskite interface as the perovskite conversion process starts from the top and then penetrates into the PbI_2_ bulk under fast conversion conditions [5,33]. This quick process creates the perovskite barrier between the salts and the PbI_2_, which is presented schematically in Figure 1b. The unreacted salt can be managed via evaporation, leaving behind the unreacted PbI_2_. Although, a moderate amount of PbI_2_ residue is advantageous for the perovskite, if it lies within the grain boundaries. At the interfaces, PbI_2_ has adverse effects in enhancing the nonradiative recombination and thus retards the performance of the devices.

**Figure 2:**
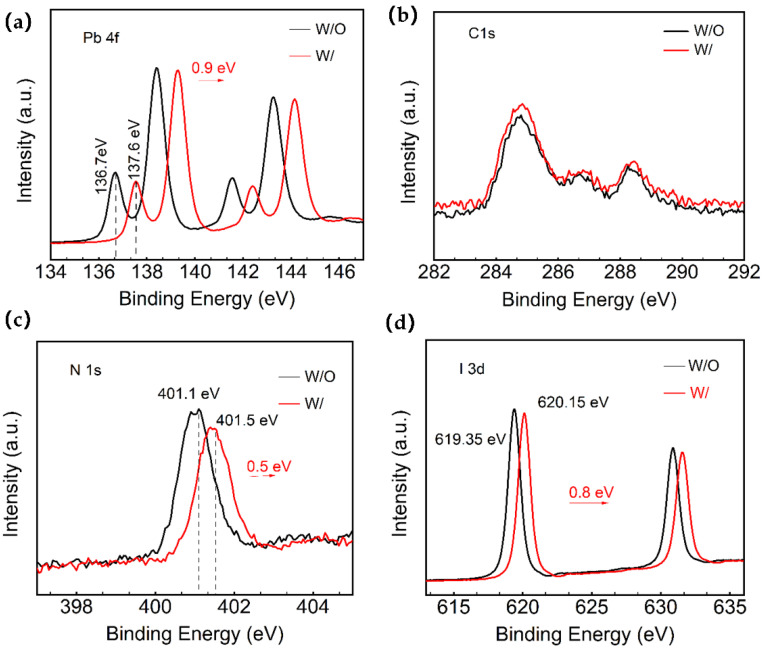
XPS spectra of the control group and experimental group: (**a**–**d**) peak position change charts for Pb4f, C1s, N1s, and I3d.

X-ray diffraction (XRD) without (W/O) ART and with (W/) ART-deposited perovskite is conducted to study the effect of ART on the PbI2 residue and the perovskite defects’ passivation. Improvement in the crystallization has been noticed, and the peak of PbI_2_ is substantially reduced (Figure 1d). Specifically, the Bragg peak at the 2theta = 12.6° position, which represents the PbI_2_, is suppressed under the effect of ART (Figure 1e). At the X-axis, the diffraction peaks of the W/ and W/O perovskite samples are approximately the same, indicating that ART does not affect the perovskite’s crystal lattice (Figure 1e, Supporting Figure S1a,b). Furthermore, the perovskite crystallinity is enhanced in the perovskite deposited on ART, which can be seen from the sharpness of the W/ peaks (Figure 1d). For obtaining information on the surface morphology of the perovskite film, a scanning electron microscope (SEM) and atomic force microscopy (AFM) characterization tests are performed (Supporting Information, Figure S2a,b). Figure 2c,f shows the top-view SEM images of the perovskite thin films, which show only a slight improvement in the film smoothness, coverage, and grain sizes. Modification of the perovskite layer via ART led to a significant reduction in the perovskite film’s roughness, as observed in the AFM. Root mean square (RMS) analysis revealed that the perovskite deposition on ART reduced the roughness of the perovskite film from 42.67 nm to 35.61 nm.

Moreover, the Pb^2+^ defects in the W and W/O ART perovskite samples are analyzed via X-ray photoemission spectroscopy (XPS). Figure S3 shows the full XPS spectrum. From the XPS spectra of Pb4f in Figure 2a, it can be noticed that the two prominent peaks of Pb4f (4f7/2: 138.45 eV/139.15 eV and 4f5/2: 143.25 eV/144.15 eV) are seen in both the W and W/O ART perovskite samples. In particular, the small peaks of Pb^2+^ corresponding to the saturated coordination and low binding energy (4f7/2: 136.7 eV/137.6 eV and 4f5/2: 141.6 eV/142.5 eV) were attributed to the uncoordinated metallic Pb (Pb^0^). These findings suggest that the use of ART at the perovskite interface could impact the chemical composition of the perovskite films. The Pb 4f spectrum of both the control group and the ART/perovskite films showed the presence of metallic lead, indicating the reduction in Pb^2+^ to Pb^0^. The Pb 4f7/2 and Pb 4f5/2 binding energies of the experimental group were significantly shifted towards a higher binding energy by 0.9 eV compared to the control group. These results indicate the effective passivation of the perovskite defects around the unreacted PbI_2_ sites, as the small peak of low binding energy decreased in intensity and shifted by 0.9 eV towards a higher binding energy. Additionally, the introduction of the ART layer effectively passivates the Pb-related surface defects and stabilizes the surface dangling bonds, as supported by the attenuation of the Pb^0^ peak in the sulfur compounds [34,35].

Moreover, the obtained spectra exhibited characteristic perovskite crystal peaks such as C1s, N1s, O1s, Pb4f, and I3d (Figure S3). Fine scan spectra of C1s, N1s, O1s, Pb4f, and I3d were obtained under different X-ray irradiation conditions before the measurements. The detailed Pb4f, C1s, N1s, and I3d spectra of the films are depicted in Figure 2a–d. The results indicated a slight shift in the peak positions towards a higher binding energy in the films deposited on ART. The split-peak fits of I3d, Pb4f, and C1s for the control and the ART-based films can be found in Supporting information Figure S4, with the corresponding elemental contents in Table S1. The effects of this study on the Pb defects via XPS analysis is supported by the previous finding of Tsutomu Miyasaka’s team [28]. They demonstrated that the strong interaction between the ART molecules and the uncoordinated Pb^2+^ ions inhibits the formation of new Pb clusters with deep capture defects during aging, as indicated by the theoretical and experimental calculations. These findings highlight the potential of ART as a passivation layer for improving the stability and performance of devices.

**Figure 3:**
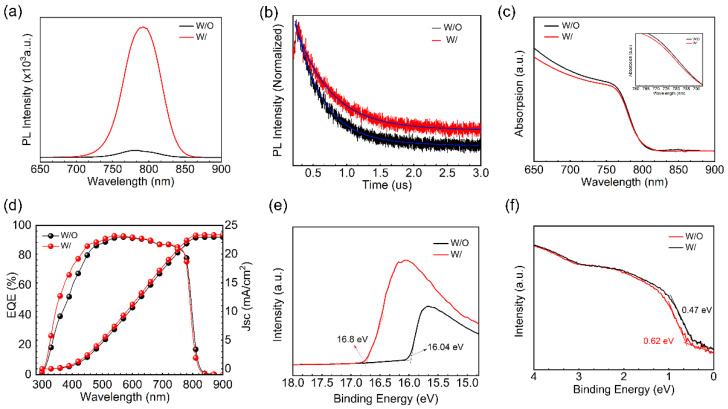
(**a**) Comparison of PL peaks between the control group and experimental group; (**b**) comparison of PL decay curves between the W and W/O ART; (**c**) comparison of UV-Vis absorption spectra and the enlarged image of the blue shift position of the signal peak between the W and W/O ART; (**d**) EQE spectra of the W/ and W/O ART layer; (**e**) secondary electron cut-off region (SECO) taken from UPS spectra; and (**f**) valence band region with logarithmic intensity scale taken from UPS spectra.

**Figure 4:**
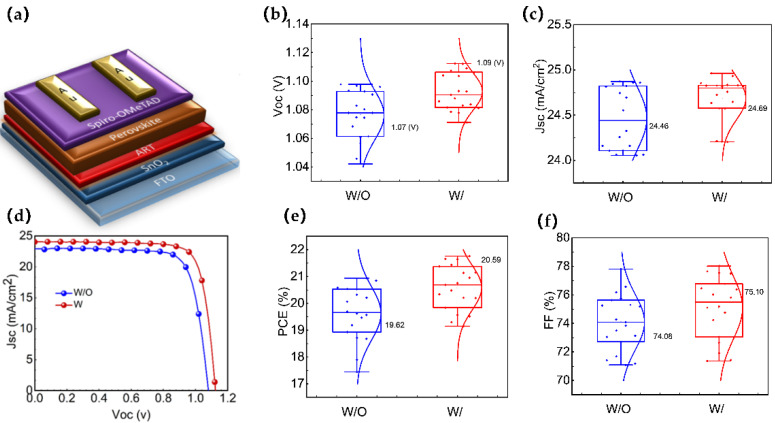
(**a**) device structure; (**b**,**c**) statistical comparison of J_SC_ and V_OC_ between the control and ART-based devices, average values are mentioned (based on 30 devices); (**d**) I-V curves of control and ART-based PSC; and (**e**,**f**) statistical comparison of PCE, and FF between the control and ART-based, average values are mentioned (based on 30 devices).

Time-resolved photoluminescence (TRPL) measurements are performed to investigate the optical and carrier properties of the perovskite films for both samples (W/ and W/O ART). The results indicate that the PL intensity in Figure 3a, the ART/perovskite film, is significantly enhanced, and a red-shift phenomenon was noticed. The ART/perovskite films exhibited an increase in the carrier lifetime, as shown in Figure 3b (fitting parameters are included in Table S2), suggesting that the inclusion of the ART layer effectively improved the quality of the perovskite films by reducing the PbI_2_ residue and the Pb^2+^ defects, which alternatively decreased the non-radiative recombination. The extension of the relaxation time resulting from the ART/perovskites indicates that the duration of the band-to-band recombination emission was prolonged, leading to the conclusion that the ART passivation layer decreased the density of the shallow traps. We utilized TRPL spectrum fitting, which revealed a remarkable improvement in the lifespan of the ART/perovskite film. Specifically, the ART/perovskite film exhibited a lifespan of 509.93 ns, which is significantly longer than that of the control sample (394.92 ns). These promising results can be attributed to the reduction in PbI_2_ at the perovskite interface. Additionally, reductions in the non-radiative recombination further resulted in improved device stability. Notably, the modified perovskite film exhibited an enhanced long-term stability of the conversion efficiency. Overall, these findings suggest that the reduction in the PbI_2_ residue at the interfaces has the potential to improve the performance of PSCs. The results of this study provide valuable insights into the mechanisms underlying the enhanced performance of perovskite solar cells with the ART passivation and PbI_2_ reduction. We compared the absorption changes of the samples with and without an ART layer using UV-vis measurements. We found that the absorption edge of the perovskite film shifted slightly blue after the treatment with the artemisinin passivation layer. The same phenomenon was observed in the EQE measurements. This indicates that the perovskite defects were passivated and the PbI_2_ residue is suppressed, thereby reducing the non-radiative recombination. In Figure 4d, the integrated current densities of 22.96 mA/cm^2^ and 23.33 mA/cm^2^ for the external quantum efficiency (EQE) spectra of the W/O ART layer and ART passivation layer devices, respectively, are in good agreement with the J-V measurements.

The ART layer greatly affects the optical and electronic properties of the perovskite films. Ultraviolet photoelectron spectroscopy (UPS) and the photoluminescence (PL) absorption peaks were used to analyze these properties before and after the ART application (Figure 3e,f). It is evident that the defect passivation of perovskite promotes a charge extraction and alters the energy levels accordingly [36]. In this virtue, the ART layer’s influence on the energy scape of perovskite is studied via UPS. The results indicate that the ART layer slightly decreases the secondary electron cutoff region (SECO) (Figure 3e,f), and there is a change in the valence band maximum (VBM) of the perovskite films (Figure S5). Specifically, the work function (WF) values after modification were 4.42 eV for the control and 5.18 eV for the ART/perovskite. Moreover, the VBM values for the ART modified and control perovskite films were found to be 0.62 eV and 0.47 eV below the Fermi level (E_F_), respectively. These changes in the energy levels of the perovskite films indicate a preferred arrangement of energy levels that may benefit the charge transfer and non-radiative composite suppression. Therefore, compared with the perovskite treated without the ART layer, the energy level of the perovskite treated with the ART passivation layer is more matched, which favors carrier extraction and increases the efficiency of the device.

The final device structure is schematically presented in Figure 4a, and the JV curves of the best devices for both W/ and W/O are shown in Figure 4b. The maximum efficiency of the perovskite solar cells increased from 20.21% for the devices without the ART layer, with a Jsc of 24.86 mA/cm^2^, Voc of 1.07 V, and an FF of 0.76, to 21.72% for the devices with the ART layer, with a Jsc of 24.69 mA/cm^2^, Voc of 1.11 V, and an FF of 79.52 (Champion devices’ photovoltaic parameters for both types are included in Table S3). We investigated the performance reproducibility of the ART-based PSCs by assessing the photovoltaic parameters of 30 devices for each type (W and W/O ART). The photovoltaic parameters (Jsc, Voc, FF, and PCE) are evaluated, as shown in Figure 4b,c,e,f. Remarkably, all of the photovoltaic parameters improved after the inclusion of the ART layer. The calculated average Voc, Jsc, FF, and PCE values in the W/O ART devices were 1.07 V, 24.46 mA/cm^2^, 74.08, and 19.62%, respectively. On the contrary, in the W/ ART devices, the average values of Voc, Jsc, FF, and PCE were 1.09 V, 24.69 mA/cm^2^, 75.10, and 20.51%, respectively. In addition, the hysteresis index (HI) was determined to quantify the efficiency mismatch using the equation: HI = (PCE_reverse_ − PCE_forward_)/PCE_reverse_. The HI decreased from 0.64% for the devices without the ART layer to 0.41% for the devices with the ART layer interface modification (Table S3), indicating a reduction in the defects and hysteresis effect. These results demonstrate that the ART passivation layer significantly improves the performance and stability of perovskite solar cells.

**Figure 5:**
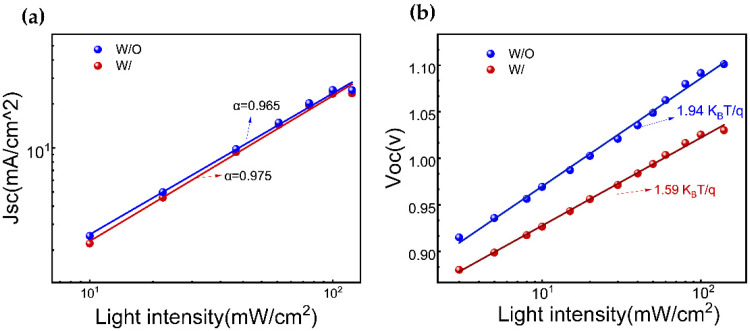
(**a**) The function relationship between (**a**) Jsc and (**b**) Voc with incident light intensity for W/O and W/ ART device.

Furthermore, the charge recombination dynamics in a device are investigated by analyzing the functional relationships of short-circuit current density (Jsc) (Figure 5a) andthe open-circuit voltage (Voc) (Figure 5b)with incident light intensities. The ideal factor (α) of the device, calculated as (α ≤ 1), is found to be 0.965 and 0.975 for W/O and W/ ART, respectively, indicating that the bimolecular recombination approaches the minimum value, especially in ART-based devices. On the other hand, the ideal factor (ε) under the open-circuit conditions, with a range of 1 < ε < 2, reflects the extent of the monomolecular recombination. A value of ε closer to 1 indicates lesser monomolecular recombination. In contrast, a value more relative to 2 suggests more traps in the device, leading to increased monomolecular recombination, which is unfavorable for the charge transmission and collection. The experimental results reveal that the ε values of the devices are all greater than 1, indicating the existence of monomolecular recombination. As expected, on the ART incorporation, ε value is reduced from 1.94 K_B_T/q to 1.59 K_B_T/q. These findings are consistent with the results obtained from the TRPL and UV-visible absorption spectroscopy measurements. Consequently, these results are the response of the passivation treatment, and especially, the PbI_2_ residue reduction via ART.

## 3. Materials and Methods

### 3.1. Materials

The SnO_2_ colloidal dispersions, featuring a particle size of 3–5 nm and diluted with 15% water, were obtained from Alfa Aesar. Meanwhile, flourine–doped tin oxide (FTO) with a R_sheet_ of 15 Ω/sq, thickness of 600 nm, and a light transmittance of >85% was procured from Beijing Huamin New Material Technology Co., Ltd., Beijing, China. Lead iodide (PbI_2_), MAI, FAI, MACl, MABr, and Spiro-OMeTAD were sourced from Xi’an Shuoyuan Optoelectronic Technology Co., Ltd., Xian, China, and supporting reagents such as K_2_SO_4_, triethylsilane, N, N-dimethylformamide (DMF), dimethyl sulfoxide (DMSO), chlorobenzene (CB), 4-tert-butylpyridine (TBP), anhydrous isopropanol (IPA), and lithium bisfluoromethane sulfonamide (Li-TFSI) were purchased from Sigma Aldrich Co., Ltd., St. Louis, MO, USA. Artemisinin was also procured from Sigma.

### 3.2. Device Fabrication

Substrate Preparation: The FTO conductive glass substrate was sequentially cleaned with a glass cleaner, ultrapure water, isopropanol, and ethanol for 15 min each, and then dried with a nitrogen gun for use. A two-step process was used to prepare the n-i-p perovskite solar cells with the FTO/SnO_2_&18C6&K/artemisinin passivation/perovskite/Spiro-OMeTAD/Au structure. The prepared FTO substrate was pretreated with UV-ozone for 15 min to ensure surface infiltration and establish good contact with SnO_2_.

In the context of the electron layer preparation, a precursor solution composed of SnO_2_ with a colloidal volume ratio of 15% and ultrapure water containing 0.2 mg of 18C6 and 2 mg of K_2_SO_4_ was combined at a ratio of 1:5. Following a 15 min sonication period, 100 uL of the electron layer precursor solution was subjected to a spin-coating process at 4000 rpm for 30 s, depositing a layer on the previously ozone-treated FTO substrate. The resultant film was annealed at 150 °C for 15 min, resulting in a dense SnO_2_ electron layer that was allowed to cool to room temperature before being exposed to an additional 15 min ozone pretreatment. The film was rapidly transferred to a glove box for subsequent spin-coating. This process was regarded as a vital component of the electron layer preparation for the intended perovskite solar cell structure.

The passivation layer was prepared by dissolving 1 mg of artemisinin in 1 mL of DMSO and subsequently deposited on the previously UV-treated SnO_2_ electron layer film using a spin-coating process at 5000 rpm for 30 s. The concentration of ART in DMSO is optimized after trying different ratios in DMSO (Supporting Information, Table S4). This preparation process is deemed critical for the successful fabrication of the intended perovskite solar cell structure.

A two-step method was utilized for the preparation of the perovskite layer films. The precursor solution (1)containing 1.5 M of PbI_2_, was dissolved in a mixture of DMF and DMSO (with a volume ratio of 9:1) and stirred at 60 °C for 4 h. The ammonium salt precursor solution (2) was composed of FAI, MABr, and MACl at a ratio of 90 mg:9 mg:9 mg and dissolved in 1 mL of anhydrous isopropanol. To deposit the perovskite layer, 60 μL of the prepared precursor solution (1) was spin-coated at 1500 rpm for 30 s, followed by annealing at 70 °C for 1 min, cooling to room temperature, and the subsequent deposition of 120 μL of the precursor solution (2) at 2000 rpm for 30 s with a quick drop at 5 s. The perovskite film was transferred to a drying air oven at 20–30% humidity for 15 min, annealed at 150 °C, and then swiftly moved to a nitrogen glove box. It was allowed to cool to room temperature and was then ready for the next step.

The hole precursor solution was formulated by dissolving 72.3 mg of Spiro-OMeTAD doped with 17.5 μL Li-(TFSI) in a 1 mL acetonitrile solution at a concentration of 520 mg, and 28.8 μL of 4-tert-butyl pyrimidine was added to the solution. The resulting 60 μL hole precursor solution was spin-coated and deposited onto the perovskite films at a rate of 4000 rpm for 30 s. These findings provide useful insights into the design and optimization of perovskite solar cells.

### 3.3. Photovoltaic Measurement

The manuscript reports the comprehensive characterization of a perovskite solar cell device. The current density–voltage (I-V) curve of the device was measured using a Keithley B2901A digital source (Keithley Instruments, Inc., Cleveland, OH, USA) meter under AM1.5G solar simulator irradiation, and the incident light intensity was calibrated with a quasi-silicon cell. The I-V curve was recorded with a scanning step of 0.02 V, a delay of 10 ms, and an effective area of 0.06 cm^2^. Time-resolved photoluminescence (Edinburgh Instruments, Bain Square Kirkton Campus Livingston EH54 7DQ, Britain, UK) spectra were acquired using a transient steady-state fluorescence lifetime test (FluoTime 300, Picoquant, Berlin, Germany). The surface roughness of the perovskite films was determined via atomic force microscopy (AFM) (Oxford Instruments Asylum Research, Inc., Santa Barbara, CA, USA). The morphology of the film surface and sections was analyzed using field emission scanning electron microscopy (SEM) (GeminiSEM 300 Carl Zeiss Microscopy Ltd., Britain, UK). Fourier transform infrared spectra were obtained using a Nicolet iS50, Thermo Fisher Scientific, Co. Ltd., Brno-Černovice, Czech Republic. X-ray diffraction (XRD) was performed using a SmartLab XRD, Rigaku Corporation of Japan, Tokyo, Japan. XPS Escalab Xi+, Thermo Fisher Scientific Co. Ltd., Brno-Černovice, Czech Republic. The TR-PL spectra had a transient lifetime of 30 ms to 20 ps and a wavelength range of 230 nm to 1700 nm. These results provide valuable insights into the structural and optical properties of the perovskite solar cell, which could help in the development of high-performance devices.

## 4. Conclusions

Addressing the defects related to the perovskite layer, which are generated via the two-step method, will speed up the commercial deployment of PSCs, as this method is comparatively more appealing to industries as compared to the anti-solvent process. PbI_2_ residue-related issues are one of the top ill-defined issues as some believe it helps in the defects’ passivation while others believe it hinders the photovoltaic performances. In this work, the ART molecule is applied at the interface of the SnO_2_/perovskite, and it was notified that the PbI_2_ residue was reduced, where a defect-free perovskite layer was obtained. Two essential molecular moieties of ART, the ketonic group and alkyl chains, helped in the Pb^2+^ passivation and enhanced the hydrophobicity. Therefore, efficiency and stability are both improved in the ART-based PSC devices. The modified devices retained more than 95% of their initial PCE when stored in the N2 environment (at 25 °C) (Supporting Information, Figure S6). Additionally, after the in-depth experimentation and testing analysis, it can be concluded the PbI2 residue hinders the performance at the interfaces and gives rise to the Pb^2+^ defects.

## Data Availability

Not applicable.

## Supplementary Materials

The following supporting information can be downloaded at: https://www.mdpi.com/article/10.3390/molecules28207120/s1, Figure S1: Peak positions for different phases in the control and experimental group, including (PbI_2_) and (001), (110), and (012) phases; Figure S2: AFM spectra of (a) control and (b) the ART/perovskite layer; Figure S3: Full XPS spectrum of control and modified perovskite layer; Figure S4. XPS spectra of the control group and experimental group. (a) Peak fitting of I3d peak position change; (b) Peak fitting of Pb4f peak position change; (c) Peak fitting of C1s of the control group (perovskite thin film without artemisinin passivation layer modification); (d) Peak fitting of C1s of the experimental group (perovskite thin film with artemisinin passivation layer modification); Figure S5: Based on intercepts of the VB region (EB, min) and the SECO region (EB, max) (a) Energy levels and fermi levels (calculated as-(21.22-EB, max) eV; the VBM levels were calculated as - (21.22 - EB, max + EB, min)) diagrams of perovskite (W/O and W/ ART) (b) The energy levels of all the layers of the device; Figure S6: Normalized PCE of the corresponding devices (W/ and W/O ART) kept in N2-glove box (at 25 °C) for 550 h; Table S1: XPS analysis was used to determine the percentage of surface chemical elements in the control group and the artemisinin passivation layer modified perovskite thin film; Table S2: The PL decay curves of the control group and the perovskite thin film modified with artemisinin passivation layer; Table S3: The photoelectric parameters of the control group and the perovskite thin film modified with artemisinin passivation layer; Table S4: The photoelectric parameters of the control group and the modified with different concentrations of artemisinin in DMSO.
