# Supplementary material for: A Functional Biological Molecule Restores the PbI2 Residue-Induced Defects in Two-Step Fabricated Perovskites
Source: Molecules. 2023 Oct 17;28(20):7120. doi: 10.3390/molecules28207120 (PMC10608809; doi:10.3390/molecules28207120)
Supplement: Supplementary file 1 [file molecules-28-07120-s001.zip › molecules-2564513-supplementary.pdf]

molecules

Supporting Information

A functional biological molecule restores the PbI_2 residue-induced defects in two-step fabricated perovskites

Yuanmei Huang [1,2], Guoping Yu [2], Danish Khan [2], Shuanglin Wang [3], Yujie Sui [3], Xin Yang [1], Yu Zhuang [1], Jun Tang [2], Huaxi Gao [1], Ming Xin [1], Abuduwayiti Aierken [1,*] and Zeguo Tang [2,*]

[1] School of Energy and Environment, Yunnan Normal University, Juxian Road 768, Chenggong, Kunming 650500, China
[2] College of New Materials and New Energies, Shenzhen Technology University, Lantian Road 3002, Shenzhen 518118, China
[3] College of Materials Science and Engineering, Beijing University of Technology, 100 Pingleyuan, Beijing 100124, China
* Correspondence: erkin@ynnu.edu.cn (A.A.); tangzeguo@sztu.edu.cn (Z.T.)

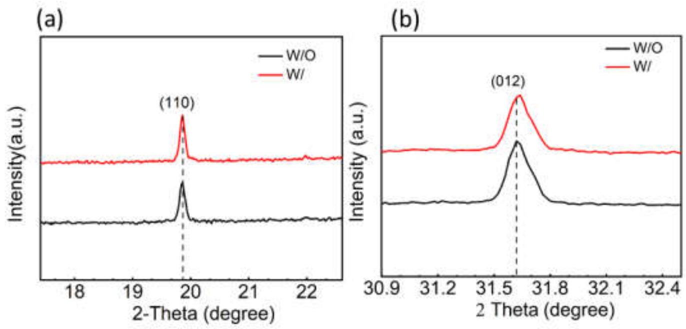

Figure S1: Peak positions for different phases in the control and experimental group, including (PbI_2) and (001), (110), and (012) phases;

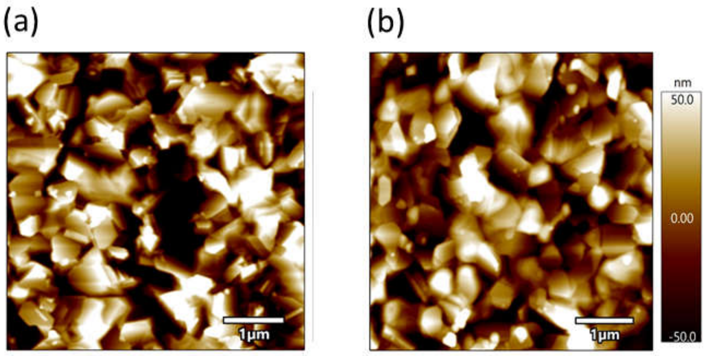

Figure S2: AFM spectra of (a) control and (b) the ART/perovskite layer;

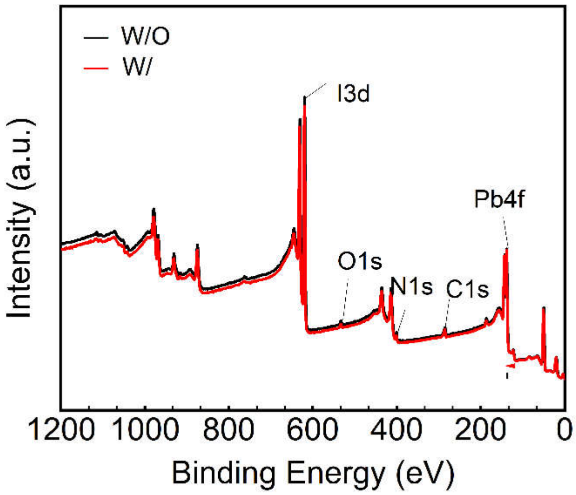

Figure S3: Full XPS spectrum of control and modified perovskite layer;

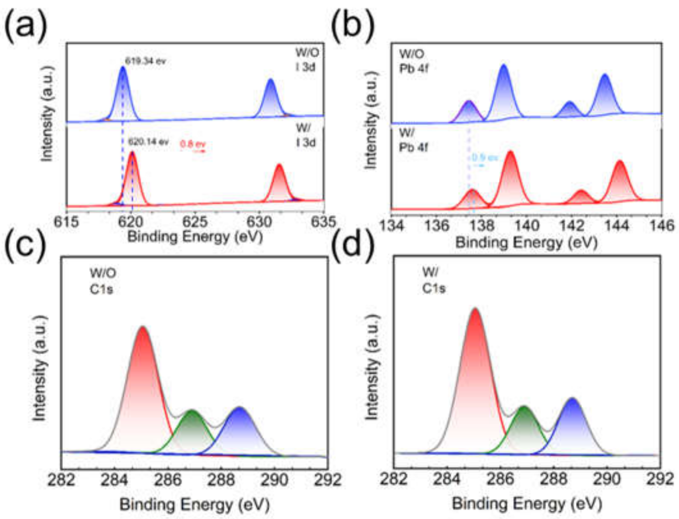

Figure S4: XPS spectra of the control group and experimental group. (a) Peak fitting of I3d peak position change; (b) Peak fitting of Pb4f peak position change; (c) Peak fitting of C1s of the control group (perovskite thin film without artemisinin passivation layer modification); (d) Peak fitting of C1s of the experimental group (perovskite thin film with artemisinin passivation layer modification);

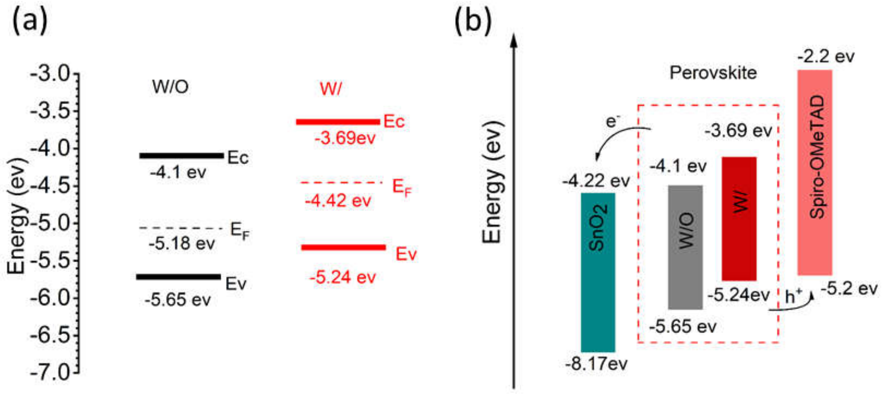

Figure S5: Based on intercepts of the VB region (EB, min) and the SECO region (EB, max) (a) Energy levels and fermi levels (calculated as - (21.22 - EB, max) eV; the VBM levels were calculated as - (21.22 - EB, max + EB, min)) diagrams of perovskite (W/O and W/ ART) (b) The energy levels of all the layers of the device;

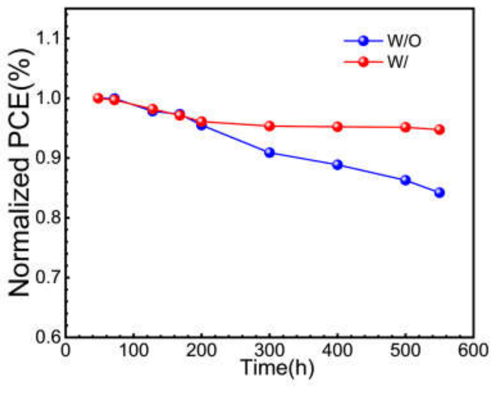

Figure S6: Normalized PCE of the corresponding devices (W/ and W/O ART) kept in N2-glove box (at 25 °C) for 550 h;

Table S1: XPS analysis was used to determine the percentage of surface chemical elements in the control group and the artemisinin passivation layer modified perovskite thin film;

Sample	Pb4f	C1s	O1s	N1s	I3d
W/O	13.96	31.21	6.21	12.43	36.36
W/	13.86	31.88	5.88	10.84	36.19

Table S2: The PL decay curves of the control group and the perovskite thin film modified with artemisinin passivation layer;

Sample	τ_1	A_1	τ_2	A_2
W/O	394.92	1643.05	2823.17	116.42
W/	509.93	1233.79	565943.52	2443.20

The Table S2 values are measured by using ($\boldsymbol{y} = \boldsymbol{A_1 exp}^{\left(-\frac{x}{\tau_1}\right)} + \boldsymbol{A_2 exp}^{\left(-\frac{x}{\tau_2}\right)} + \boldsymbol{y_0}$).

Table S3: The photoelectric parameters of the control group and the perovskite thin film modified with artemisinin passivation layer;

Sample	Scan direction	J_{sc} (mA/cm^2)	V_{oc} (V)	FF	PCE (%)
W/O	Forward	24.45	1.08	75.81	20.08
	Reverse	24.86	1.07	75.99	20.21
W/	Forward	24.84	1.11	78.81	21.63
	Reverse	24.69	1.11	79.52	21.72

Table S4: The photoelectric parameters of the control group and the modified with different concentrations of artemisinin in DMSO;

ART ratio in DMSO	Scan direction	Voc (V)	FF (%)	Jsc(mA/cm^2)	PCE (%)
0mg/ml	Forward	1.08	75.81	24.45	20.08
	Reverse	1.07	75.99	24.86	20.21
1mg/ml	Forward	1.11	78.81	24.84	21.63

	Reverse	1.11	79.52	24.69	21.72
1.4mg/ml	Forward	1.10	77.33	24.26	20.69
	Reverse	1.10	76.74	24.26	20.49
2mg/ml	Forward	1.02	77.26	24.57	19.42
	Reverse	1.01	75.42	24.42	18.55
